# Mixed Small Vessel Disease in a Patient with Dementia with Lewy Bodies

**DOI:** 10.3390/brainsci9070159

**Published:** 2019-07-04

**Authors:** George P. Paraskevas, Vasilios C. Constantinides, Efstratios-Stylianos Pyrgelis, Elisabeth Kapaki

**Affiliations:** 1st Department of Neurology, Cognitive and Movement Disorders clinic and Unit of Neurochemistry and Biological Markers, School of Medicine, National and Kapodistrian University of Athens, Eginition Hospital, 10679 Athens, Greece

**Keywords:** cerebral amyloid angiopathy, subcortical small vessel disease, dementia with Lewy bodies, amyloid beta, cerebrospinal fluid biomarkers

## Abstract

Background: Cerebral amyloid angiopathy (CAA) is characterized by deposition of amyloid in small/medium size brain vessels, and may coexist with Alzheimer’s disease or dementia with Lewy bodies (DLB). We describe a patient with a clinical diagnosis of DLB and imaging/biochemical characteristics suggestive of mixed small vessel disease (both CAA and non-amyloid microangiopathy). Methods: Clinical evaluation according to recent diagnostic criteria, magnetic resonance imaging, dopamine-transporter scan (DAT-scan) and cerebrospinal fluid (CSF) analysis for dementia biomarkers were all performed. Results: The patient is a 71-year-old male, fulfilling criteria for probable DLB, with a positive DAT-scan, but with multiple microbleeds in a cortical-subcortical location suggestive of CAA, some microbleeds in deep brain nuclei suggestive of non-amyloid microangiopathy and abnormal levels of only amyloid-beta (Aβ_42_) in CSF. Conclusion: Coexistent mixed vascular and neurodegenerative disorders are frequent in older subjects with dementia and each one of the underlying pathologies may contribute to, or modify the clinical presentation.

## 1. Introduction

Cerebral amyloid angiopathy (CAA) comprises a heterogeneous group of disorders characterized by deposition of amyloid, primarily in leptomeningeal vessels as well as medium or small size vessels of the cortex (particularly small arteries, arterioles and capillaries). Various causes may result in CAA, depending on the amyloid protein involved [1]. The resulting pathology of the vessel wall may lead to hemorrhagic complications (such as lobar hemorrhage, focal/convexal subarachnoid hemorrhage, cortical/corticosubcortical microbleeds and cortical superficial siderosis) and/or ischemic complications including white matter ischemic lesions or cortical (micro)infarcts [1]. Amyloid beta (Aβ) deposition is an important cause of CAA. This may occur (a) sporadically, either alone (particularly in the elderly) or in conjunction with sporadic Alzheimer’s disease (AD) and (b) in cases of hereditary AD or CAA, which may include mutations in Presenilin 1 and 2, as well as in Amyloid Precursor Protein [2]. A significant percentage of AD patients (up to 82%–98%) exhibit pathologically some degree of CAA, 30% of which may be severe [3,4,5].

Dementia with Lewy Bodies (DLB) is clinically characterized by frontal-subcortical types of dementia, parkinsonism and various combinations of fluctuations in cognition, visual hallucinations and other psychotic features, neuroleptic sensitivity and rapid eye movement (REM) sleep behavior disorder, and this clinical picture is different from the amnestic type of dementia usually seen in AD [6]. From the pathological/biochemical point of view, DLB is characterized by abnormal aggregation of α-synuclein within neurons and thus, it is considered as a synucleinopathy, much like Parkinson’s disease (PD) [7]. Despite being synucleinopathies, concomitant extracellular Aβ deposition and even vascular or full-blown AD pathology, are found not uncommonly in DLB and less frequently in PD [8]. Thus, it is not surprising that pathologically proven CAA has been described in up to 50% of patients with DLB [9].

We present a patient with a clinical diagnosis of DLB and imaging/biochemical characteristics suggestive of CAA in addition to non-amyloid microangiopathy.

## 2. Materials and Methods

### 2.1. Study Details

The patient was routinely hospitalized in a tertiary reference academic center. Prior to participation, the patient and his wife gave informed consent for inclusion in the “Migraine and Specific Vasculopathies Registry/Study” of the 1st Department of Neurology, National and Kapodistrian University of Athens, Greece, which is performed according to the 1975 Declaration of Helsinki, revised in 2013 and has been approved by the Scientific and Ethics Committee of Eginition Hospital (approval 277/27-7-2011).

After complete physical and neurologic examination, serum biochemical analysis, neuroimaging and cerebrospinal fluid (CSF) analysis were performed. For the diagnosis of Dementia with Lewy Bodies (DLB) and vascular cognitive disorders, the more recent recommendations (4th consensus report) of the DLB Consortium [6] and the International Society for Vascular, Behavioral and Cognitive Disorders (VASCOG) statement diagnostic criteria [10] were used respectively.

### 2.2. Neuroimaging

Magnetic resonance imaging (MRI) was performed on a 3T scanner (Magnetic Philips Medical Systems—Achieva 3.0 T (TX), Amsterdam, the Netherlands). The sequences included T1-weighted axial, sagittal, and coronal images, 3D Fluid Attenuation Inversion Recovery (FLAIR) images, axial proton density and T2 images, axial Diffusion Tensor Imaging (DTI) and susceptibility weighted imaging (SWI). Additionally, dopamine transporter scan (DAT-scan) by single photon emission computerized tomography was performed using 185 MBq 123I-FP-CIT (GE Healthcare).

### 2.3. CSF Sampling and Biomarker Analysis

Lumbar puncture was performed at 11:00 a.m., after overnight fasting, at the L4–L5 interspace, according to recently proposed recommendations on standardized operating procedures (SOPs) for CSF biomarkers [11]. CSF was collected in four tubes. The first two tubes (2mL each) were used for cytology, biochemistry, IgG index, syphilis serology and oligoclonal bands determination. The remaining two tubes (5ml each) were aliquoted in propylene tubes (750 μL each) after immediate centrifugation and were stored at −80 °C pending analysis. Aliquots were thawed once, before analysis which was performed 2 months after collection and storage.

The CSF levels of total tau protein (τ_T_), amyloid-β peptide (1–42) (Aβ_42_) and tau phosphorylated at threonine-181 (τ_P-181_) were measured in duplicate, blindly, by double sandwich, enzyme-linked immunosorbent assay (ELISA) by use of commercially available kits (“Innotest hTau antigen”, “β-amyloid1-42” and “phosphor-tau 181” respectively, Fujirebio, Gent, Belgium), in accordance with instructions by the manufacturer. A 4-parameter logistic curve was applied for determinations. Inter-assay and intra-assay variation of <6.6% and minimal measurement error (≤3.3%) were achieved by use of in-house standards during runs [12]. For abnormality, current cut-off values of our laboratory (τ_T_ ≥ 376, Aβ_42_ ≤ 580 and τ_P-181_ ≥ 62.5 pg/mL) were used, based on a large sample of healthy controls and those with Alzheimer’s disease [13].

## 3. Results

### 3.1. Patient Description

The patient was a 71-years-old male, with no personal history of smoking, hypertension or other cardiovascular risk factors and no significant family history. At age 68 he gradually developed significant mental slowness, apathy, attention and concentration difficulties, visual hallucinations and gait difficulty with slow and short steps. During the following 2–3 years both cognition and gait deteriorated. Fluctuating attention and day-time episodes of sleepiness were additionally present. At least two episodes of symptomatic orthostatic hypotension were reported, as well as limb movements and talking during sleep, compatible with REM sleep behavior disorder.

Neurological examination revealed significant symmetric parkinsonism with lead-pipe and cogwheel rigidity, severe bradykinesia and absence of tremor, bilateral pyramidal signs with extensor plantar reflexes, primitive reflexes and significant gait difficulty. Cognition was severely affected and neuropsychological testing could not be performed, except for the Mini-Mental State Examination (MMSE) [14] with a score of 2/30. During his hospitalization significant fluctuations of arousal were observed between days, or even during the same day.

Routine hematology and serum biochemistry were within normal limits, including coagulation testing, thyroid function and levels of vitamin B12 and folate. Syphilis serology, antinuclear, anti-DNA and anti-ENA antibodies and antibodies for autoimmune or paraneoplastic encephalopathies (NMDAR, LGI1, CASPR2, GABAb1R, AMPA1R, AMPA2R, Hu, Yo, Ri, PNMA2, CV2, amphiphysin, recoverin, SOX1, Zic4, Tr, VGKC, GAD, mGluR5) were all negative.

For treatment, a rivastigmine transdermal patch (initially 4.6 mg/day) was used, resulting in improvement of arousal, reduction of fluctuations, and mild improvement of cognition (MMSE score 7/30). However, his movement disorder did not respond to levodopa.

### 3.2. Neuroimaging

**Figure 1:**
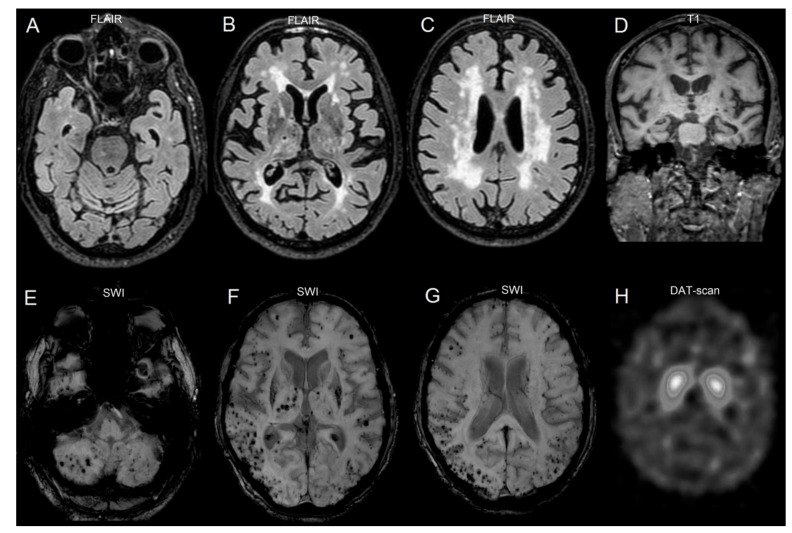
Neuroimaging of the patient. Axial Fluid Attenuation Inversion Recovery (FLAIR) images indicate white matter hyperintensities from the level of the pons up to corona radiate (**A**–**C**), while some lacunes are also present at the level of basal ganglia and thalami (B). Despite neocortical atrophy shown in B and C, coronal T1 image (**D**) indicates relative preservation of the hippocampus, but additional presence of some lacunes. Axial susceptibility weighted images (SWI) show multiple microbleeds in the cerebellum (**E**), basal ganglia (**F**) and cortical-subcortical locations with a posterior preference (**F**,**G**). DAT-scan (**H**) indicates reduction of dopamine transporter activity, with some degree of asymmetry.

On MRI, an extensive load of white matter hyperintensities, together with lacunes in basal ganglia and thalami, were present (Figure 1A–D). Multiple microbleeds were additionally observed. Some of them were located in the basal ganglia/thalami, but the main load was found in posterior corticosubcortical locations (Figure 1E–G). Some degree of cortical atrophy was observed, especially in the frontal lobes and perisylvian locations, with relative preservation of the hippocampus (Figure 1D). On DAT-scan, reduced activity of the dopamine transporter was observed (Figure 1H). 

### 3.3. CSF Analysis

Routine analysis of CSF showed normal cell count (2 lymphocytes/mm^3^ with no red blood cells), normal glucose content (68 mg/dL), but increased protein (79.2 pg/dL). Oligoclonal bands were absent and IgG index was normal (0.52, normal values <0.65), but albumin ratio was increased to 12.7 (upper normal limit 9). Biomarker analysis revealed normal τ_T_ (341 pg/mL) and τ_P-181_ (40.2 pg/mL), but decreased Aβ_42_ (385 pg/mL).

## 4. Discussion

The patient could otherwise fulfill the VASCOG criteria for probable vascular cognitive disorder [10], except for the exclusion criterion of DLB-compatible picture. On the other hand, he fulfills the 4th Consensus Criteria for DLB [6]. The presence of Subcortical small vessel disease (SSVD) in our patient does not exclude the diagnosis of DLB, but rather indicates mixed pathology [6]. Despite the absence of hemorrhagic stroke, the presence of microbleeds mainly in posterior corticosubcortical locations and in the absence of hypertension points to CAA [1]. However, according to the modified and validated Boston criteria, hemorrhagic lesions should be strictly cortical or corticosubcortical in location [15,16]. The presence of microbleeds in deep locations such as the basal ganglia and thalamus indicates that the patient does not fulfill the Boston criteria for CAA, but most likely has mixed small vessel disease, i.e., both CAA and sporadic non-amyloid microangiopathy, and the presence of lacunes supports this notion. Indeed, such mixed cases have been described and they seem to be driven mostly from classical cardiovascular risk factors such as hypertension [17,18].

Although AD is the first diagnosis to consider, DLB is the second most frequent neurodegenerative dementia coexistent with CAA [9,19]. Alzheimer’s pathology may coexist with DLB [8] and it has been suggested that the frequency of CAA in DLB increases with concomitant AD pathology [19,20]. In our patient, the CSF biomarker profile is not typical for AD, since τ_T_ and, especially, the more specific τ_P-181_, are normal [21]. However, AD cannot be excluded due to low Aβ_42_ and the pattern of cortical atrophy. On the other hand, low Aβ_42_ is not unusual for SSVD [22] and it is a typical finding in CAA [1] and in DLB [23]. Thus, the combination of clinical, imaging and biochemical data indicate the coexistence of mixed small vessel disease (including CAA and sporadic non-amyloid microangiopathy) with DLB (with or without some degree of additional AD pathology), and the presence of RBD and positive findings in DAT-scan are in favor of this in vivo diagnosis. In fact such mixed pathologies are frequent (if not the rule) in elderly patients with dementia, and each different pathology may contribute to or modify the clinical picture [24].

However, another possibility would be that CAA itself presented with a dementia resembling DLB. Not infrequently, CAA patients develop a frontal-subcortical type of cognitive impairment [25], and/or visuospatial dysfunction [26] and/or behavioral-psychiatric symptoms [27]. Indeed, in a small series of patients with CAA we have observed that patients who present in cognitive disorder departments more commonly have a non-amnestic dementia phenotype, whereas clinically evident hemorrhagic events are rare, while 17% of these patients present with DLB-like features [28].

## 5. Conclusions

The present case report adds to the notion that mixed vascular (including CAA) and neurodegenerative disorders may frequently coexist in older subjects with dementia. Each one of these underlying pathologies may modify the clinical presentation and course of the disease, or contribute with additional symptoms and signs.
